# Comment on “Sperm Injection at the Para‐Polar Body Site in Piezo‐Intracytoplasmic Sperm Injection Improves Subsequent Early Development of Bovine Embryos”

**DOI:** 10.1002/rmb2.12697

**Published:** 2025-11-17

**Authors:** Bangbei Wan, Weiying Lu

**Affiliations:** ^1^ Reproductive Medical Center, Hainan Women and Children's Medical Center Haikou China; ^2^ Department of Urology Haikou Affiliated Hospital of Central South University Xiangya School of Medicine Haikou China

**Keywords:** actin cytoskeleton, cell viability, intracytoplasmic sperm injections, oocytes, polar bodies, spindle apparatus

## Abstract

Piezo‐intracytoplasmic sperm injection (piezo‐ICSI) is increasingly used to reduce mechanical damage during sperm injection. In the recent study by Ashibe et al., placing the first polar body (PB) at the 2 or 4 o'clock position (“para‐PB piezo”) improved immediate oocyte survival versus conventional 6/12 o'clock orientation, without negatively affecting blastocyst formation or chromosomal integrity in bovine oocytes. The proposed mechanism is that drilling through the wider perivitelline gap reduces oolemma perturbation and secondary Ca^2+^ influx. We comment herein on: (1) the need for reporting effect sizes with confidence intervals; (2) the imperfect correspondence between PB orientation and spindle location and the value of combining para‐PB with spindle imaging; (3) mechanistic studies combining Ca^2+^ imaging, cortical cytoskeleton labeling, and standardized pulse logging; and (4) a recommended path toward human randomized trials with usable‐blastocyst yield and euploidy as endpoints. With these refinements, para‐PB orientation could be a low‐complexity improvement to piezo‐ICSI protocols.

Ashibe and colleagues propose orienting the first polar body (PB) at 2 or 4 o'clock for piezo‐ICSI (“para‐PB piezo”), reporting improved immediate oocyte survival versus conventional 6/12 o'clock alignment, with similar blastocyst and karyotypic outcomes in a bovine model [1]. Their interpretation—that distancing piezo pulses from the oolemma at the entry point mitigates membrane perturbation—is biologically plausible and clinically appealing.

## 1 Context and Significance

Clinical and translational data increasingly suggest that piezo‐ICSI can enhance survival/fertilization compared with conventional ICSI when executed under standardized settings [2, 3]. Moreover, classic human ICSI studies showed that the deposition site relative to the metaphase‐II spindle influences embryo development, cautioning against injections near the spindle and implying that geometry and local mechanics matter [4]. These observations frame para‐PB orientation as a low‐complexity optimization with potential clinical dividends.

## 2 Spindle–PB Relationship

A key translational issue is whether PB position safely proxies spindle location. Multiple human and mammalian studies demonstrate that PB does not reliably predict spindle position; misalignment angles can be substantial, and visualization with polarization microscopy (PolScope/Oosight) improves decision‐making [5, 6, 7]. Thus, para‐PB alignment should be seen as an ergonomic rule‐of‐thumb rather than a guarantee of spindle avoidance, and pairing para‐PB with spindle imaging may further reduce risk.

## 3 Mechanistic Plausibility (Ca^2+^ and Cortex/Oolemma Injury)

Piezo pulses transiently permeabilize the oolemma and can facilitate Ca^2+^ influx/activation, an effect well‐described in ICSI/assisted‐oocyte‐activation literature [8, 9, 10]. Orientations that minimize direct piezo impact on the membrane at tight clearances could therefore temper injury and aberrant Ca^2+^ signaling, consistent with Ashibe et al.'s graded survival effects at different distances/pulse intensities [1]. Future studies combining Fluo‐4 Ca^2+^ imaging with cortical actin markers and pulse‐dose logging could quantify this dose–response.

## 4 Methodological Clarifications to Aid Adoption

1. Report effect sizes with 95% CIs for survival, fertilization, and blastocyst endpoints to facilitate meta‐analysis and benchmarking across labs [1, 2, 3].
2. Detail tree‐like decision rules or standard operating parameters (pulse amplitude/number, pipette geometry, approach angle) so that para‐PB can be standardized in human labs [2, 3].
3. Because misclassification around category borders is common in orientation‐based protocols, specify “gray‐zones” (e.g., when PB and spindle are nearly collinear) in which spindle imaging is recommended [5, 6, 7].

## 5 From Bovine to Human ART

A patient‐level randomized within‐cycle design could compare para‐PB vs. conventional orientation in human piezo‐ICSI, with prespecified outcomes (oocyte survival, normal fertilization, usable blastocyst yield, euploidy where applicable). Given that spindle‐aligned practice and deposition‐site effects have been linked to developmental competence, integrating para‐PB with real‐time spindle visualization may deliver the clearest signal [4, 5, 6, 7].

## 6 Conclusion

Ashibe et al. provide a thoughtful, low‐overhead refinement to piezo‐ICSI that improves immediate oocyte survival in a preclinical model. Standardized reporting of effect sizes, careful attention to spindle–PB discordance, and human randomized data will be crucial to define clinical utility [1].

## Ethics Statement

The authors have nothing to report.

## Consent

The authors have nothing to report.

## Conflicts of Interest

The authors declare no conflicts of interest.

## Data Availability

The authors have nothing to report.
